# Ebstein Anomaly with Pregnancy: A Rare Case

**Published:** 2018

**Authors:** Nalini Sharma, Thiek Jion Lalnunnem, Megha Nandwani, Singh Ahanthem Santa, Baingen Warjari Synrang

**Affiliations:** 1- Department of Obstetrics and Gynaecology, North Eastern Indira Gandhi Regional Institute of Health and Medical Sciences, Shillong, India; 2- Department of Cardiology, North Eastern Indira Gandhi Regional Institute of Health and Medical Sciences, Shillong, India

**Keywords:** Ebstein anomaly, Maternal outcome, Pregnancy

## Abstract

**Background:**

Ebstein anomaly is an uncommon, complex congenital malformation of the heart with prevalence of 0.3–0.5%. It occurs in 1% of congenital heart disease cases. It is characterized by dysplastic abnormalities of tricuspid valve which involves both basal and free attachments of the tricuspid valve leaflets, with downward displacement and elongation of the septal and anterior cusp which resulting in tricuspid regurgitation, the proximal part of the right ventricle is “atrialised”, becoming thin walled and poorly contractile, along with an enlarged right atrium. With this anomaly, fertility is usually unaffected, even in women with cyanosis. The average life expectancy at birth of patients with Ebstein anomaly is 25–30 years. Due to its rarity and varied clinical presentations associated with Ebstein anomaly during pregnancy, this case was presented in this paper.

**Case Presentation:**

A 24 year old G2A1 at 39 weeks 6 days gestation with a known case of Ebstein anomaly was referred to NEIGRIHMS in April 2017 for further management as our institute is having well equipped cardiac facilities. Her antepartum period was uneventful. Elective LSCS was done at 40 weeks 3 days and a healthy baby weighing 2.5 *kg* was delivered. Intra and postpartum period was uneventful.

**Conclusion:**

Due to varied clinical presentations associated with Ebstein anomaly during pregnancy, such women should undergo close surveillance with multidisciplinary approach during the antenatal period to be diagnosed in terms of complications and hence be treated accordingly.

## Introduction

Ebstein anomaly (EA) is an uncommon, complex congenital malformation of the heart with prevalence of 0.3–0.5%. It occurs in 1% of congenital heart disease cases (1).

It is characterized by dysplastic abnormalities of tricuspid valve which involves both basal and free attachments of the tricuspid valve leaflets, with downward displacement and elongation of the septal and anterior cusp (2) resulting in tricuspid regurgitation (3), the proximal part of the right ventricle is “atrialised”, becoming thin walled and poorly contractile, along with an enlarged right atrium (4).

Patients can have a highly variable clinical course related to the anatomic abnormalities of Ebstein anomaly and their hemodynamic effects or associated structural and conduction system disease, like Atrial septal defect (ASD) (90%), pulmonary hypertension, ventricular and supraventricular tachycardia, ventricular septal defect, tricuspid atresia (30%), pulmonic stenosis and Wolf-Parkinson-White syndrome (WPW Syndrome) (up to 20% of patients) (5).

Cyanosis, pulmonary and systemic emboli, congestive cardiac failure and sudden cardiac collapse are anticipated complications (5).

Maternal mortality due to Ebstein anomaly is considered to be less than 1% in asymptomatic patients but may be as high as 5–15%, if aggravated by conditions like supraventricular arrhythmia, WPW syndrome or atrial fibrillation (4).

Due to its rarity and short life expectancy at birth, there are not many cases and varied clinical presentations associated with Ebstein anomaly during pregnancy; therefore, this case is presented to increase awareness about this entity among obstetricians.

## Case Presentation

**Figure 1.**
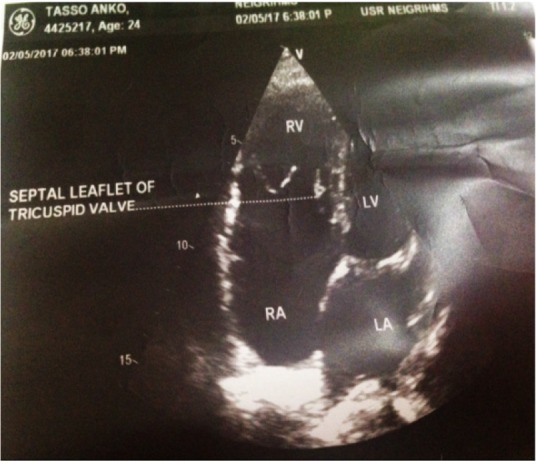
The lesion of tricuspid regurgitation with a small right ventricle

**Figure 2.**
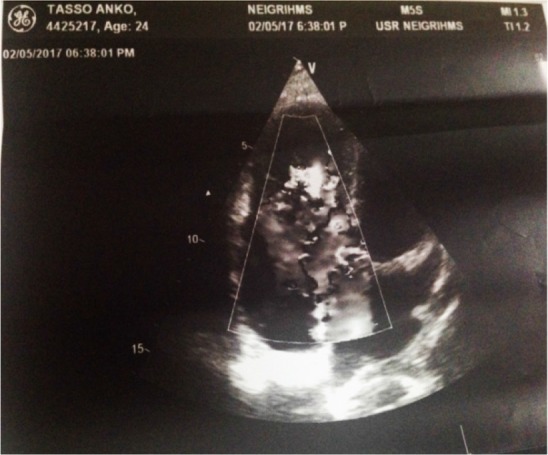
The ECHO findings of the patient. The findings are suggestive of severe tricuspid regurgitation

A 24 year old G2A1 at 39 weeks 6 days gestation with a known case of Ebstein anomaly was referred to NEIGRIHMS on April 2017 for further management as our institute is having well equipped cardiac facilities. She was diagnosed as a case of cardiac anomaly during childhood and it was confirmed by ECHO during her treatment for infertility. All these years, she did not have any symptoms related to the condition and was not on any treatment. In the present pregnancy, she had regular antenatal checkups and the whole antenatal period was uneventful. On admission, the general condition of the patient was good. No pallor, icterus, cyanosis, oedema, clubbing was seen. On examination of the respiratory system, no abnormality was detected. Her pulse rate was 90 beats per minute, regular and blood pressure was 110/80 *mm Hg* and her jugular venous pressure was normal. Her oxygen saturation in room air was 98%. On cardiovascular system examination, a split S1 was heard with audible S2, S3 and S4. Pan systolic murmur was also heard. On ECG, right axis deviation with normal sinus rhythm was seen. Echocardiography showed apical displacement of septal leaflet of tricuspid valve, elongated anterior tricuspid leaflet, dilated right atrium and right ventricle, moderate tricuspid regurgitation, small ASD and normal right ventricular function with left ventricular ejection fraction of 60% (Figures 1, 2). Echo findings confirmed diagnosis of Ebstein anomaly. Other routine investigation reports were as follows:

Blood group: O positive, haemoglobin 10.5 *gr/dl*, TLC and DLC: WNL, coagulation profile: normal, liver function tests and kidney function tests: normal, and RBS: 92 *mg/dl*. All viral markers were negative and thyroid profile was normal. Level II ultrasound obstetrics scan was done on the day of admission that showed single live intrauterine fetus with cephalic presentation; the gestational age was 37 weeks 4 days, placenta was fundo-body posterior, liquor was adequate, and expected fetal weight was 3.3 *kg*. Umbilical artery Doppler showed a normal study. No gross congenital anomaly in the fetus was observed. On per vaginal examination, her Bishop score was 4.

Cardiology opinion was taken and patient was advised to accept termination of pregnancy preferably by caesarean section to avoid prolonged induction of labor.

Elective LSCS was done at 40 weeks 3 days under epidural anaesthesia with antibiotic coverage (Injection of ampicillin 2 gram and injection of gentamycin 80 *mg* intravenously) for bacterial endocarditis prophylaxis. A healthy male baby weighing 2.5 *kg* was delivered. 10 units of oxytocin were given intramuscularly. Intraoperative and postoperative period was uneventful. After delivery, ECHO of baby was done, which was normal. Patient was discharged on the 7th postoperative day after suture removal with the advice to attend postnatal clinic and cardiology OPD after 1 month. On follow-up, patient was advised to use progester-one only pill to avoid pregnancy.

## Discussion

Half of patients with Ebstein’s anamoly were among neonates and infants with cyanosis and congestive cardiac failure. Patients who survived to adulthood may be symptomatic with onset of arrhythmia or by pregnancy. The average life expectancy at birth of patients with Ebstein anomaly is 25–30 years (6).

In Ebstein’s anomaly, there is compromised right ventricular size and function, further impaired by the increased blood volume and cardiac output during pregnancy (2). Increased right atrial pressure and volume both worsen tricuspid regurgitation. Raised catecholamine with maternal hypoxaemia and stress levels in pregnancy further predispose the cases to arrhythmia. The haemodynamic problems seen during pregnancy depend on the severity of TR and the functional capacity of the RV. Heart failure, stroke, arrhythmias, paradoxical embolism can occur even in the asymptomatic patients.

With this anomaly, fertility is usually unaffected, even in women with cyanosis (7). According to WHO, women with Ebstein anomaly without cyanosis and heart failure are categorized in class II and usually tolerate pregnancy well (8), but symptomatic patients with cyanosis and/or heart failure should be treated before pregnancy or counselled against pregnancy. While pregnant patients with EA are usually acyanotic, those with interatrial shunting can develop shunt reversal and cyanosis in pregnancy. The presence of arrhythmia or cyanosis in the mother is associated with increased maternal and fetal risk, and needs closer maternal and fetal monitoring during pregnancy and delivery (2). Mild cyanosis is associated with increased premature deliveries, low birth weight and thromboembolic complications (2, 7, 9).

The preferred mode of delivery is vaginal in almost all cases (8). During intrapartum period, one should avoid all factors leading to congestive heart failure, cyanosis and arrhythmias (10, 11). Management of patients with Ebstein’s anomaly during labor focuses on maintaining normal sinus rhythm, avoiding fluid overload and providing enough relief of pain to the patient by epidural analgesia which can be upgraded to anesthesia if cesarean section is indicated (1, 12).

The basic principles for anaesthetizing a patient with cardiac disease are maintaining both after-load and preload along with a sinus rhythm. The advantages of epidural anaesthesia are minimal intravascular volume shift, decreased catecholamine levels, control of maternal hyperventilation and most importantly, postoperative analgesia (4, 5). Intrathecal anaesthesia may complicate a right to left cardiac shunt due to sudden decrease in sympathetic vascular resistance. It is advised to cut short the second stage of labor in these patients in order to avoid the increase in intrathoracic pressure and the increase of right to left shunt (5, 10).

Large doses of oxytocin have discernible vasodilating effects and should be administered cautiously. Methylergometrine and prostaglandins increase pulmonary vascular resistance and are generally avoided. Oxytocin (5–10 *IU* intramuscularly) is commonly followed in obstetric protocols.

The present patient was lucky that she had no problem throughout the pregnancy, intrapartum and postpartum period.

Donnelly et al. discussed 42 pregnancies in 12 patients with EA. Most of them were well tolerated, resulting in 36 live births. Most of the patients were asymptomatic. Mild dyspnea in the 3rd trimester of pregnancy was common; 1 patient had paroxysmal atrial tachycardia (during the 1st pregnancy) and atrial fibrillation (during the 2nd pregnancy), and one patient had right heart failure (2). Chopra et al. described the outcome of 8 pregnancies in 4 patients with EA; one of them had right heart failure, two had arrhythmia (10).

Connolly and Warnes analyzed the outcome of 111 pregnancies in 44 women with EA (4). In this report, 16 patients were cyanotic, and 20 had an interatrial communication (ASD/PFO). Majority (76%) of pregnancies resulted in live birth, 89% were delivered vaginally, and 11% by cesarean section. The mean birth weight of infants born to cyanotic women was significantly lower than the one in newborns of acyanotic women (7).

Maternal and fetal prognosis is a good strategy in patients with Ebstein anomaly and NYHA class I. But pregnancy in Ebstein anomaly can be complicated with tachyarrhythmia or cardiac failure (13).

According to one more study, pregnant women with Ebstein anomaly are at higher chance of major adverse cardiac events like in-hospital death, acute myocardial infarction, cerebrovascular events, embolic events, cardiac complications of labor and delivery, heart failure or arrhythmia. These women have higher rate of preterm delivery, post-partum haemorrhage and LSCS (14).

## Conclusion

Maternal and fetal prognosis is favorable in patients with Ebstein anomaly and NYHA class I. But it can be complicated with various major cardiac events.

Due to varied clinical presentations associated with Ebstein anomaly during pregnancy, such women should undergo close surveillance with multidisciplinary approach during the antenatal period to be diagnosed in terms of any complications and hence to be treated accordingly.
